# Application of machine learning techniques in real-world research to predict the risk of liver metastasis in rectal cancer

**DOI:** 10.3389/fonc.2022.1065468

**Published:** 2022-12-20

**Authors:** Binxu Qiu, Xiao hu Su, Xinxin Qin, Quan Wang

**Affiliations:** ^1^ Department of Gastric and Colorectal Surgery, General Surgery Center, The First Hospital of Jilin University, Changchun, China; ^2^ Shanxi Bethune Hospital, Shanxi Academy of Medical Sciences, Tongji Shanxi Hospital, Third Hospital of Shanxi Medical University, Taiyuan, China

**Keywords:** rectal cancer, machine learning, liver metastasis, web calculator, real-world research

## Abstract

**Background:**

The liver is the most common site of distant metastasis in rectal cancer, and liver metastasis dramatically affects the treatment strategy of patients. This study aimed to develop and validate a clinical prediction model based on machine learning algorithms to predict the risk of liver metastasis in patients with rectal cancer.

**Methods:**

We integrated two rectal cancer cohorts from Surveillance, Epidemiology, and End Results (SEER) and Chinese multicenter hospitals from 2010-2017. We also built and validated liver metastasis prediction models for rectal cancer using six machine learning algorithms, including random forest (RF), light gradient boosting (LGBM), extreme gradient boosting (XGB), multilayer perceptron (MLP), logistic regression (LR), and K-nearest neighbor (KNN). The models were evaluated by combining several metrics, such as the area under the curve (AUC), accuracy score, sensitivity, specificity and F1 score. Finally, we created a network calculator using the best model.

**Results:**

The study cohort consisted of 19,958 patients from the SEER database and 924 patients from two hospitals in China. The AUC values of the six prediction models ranged from 0.70 to 0.95. The XGB model showed the best predictive power, with the following metrics assessed in the internal test set: AUC (0.918), accuracy (0.884), sensitivity (0.721), and specificity (0.787). The XGB model was assessed in the outer test set with the following metrics: AUC (0.926), accuracy (0.919), sensitivity (0.740), and specificity (0.765). The XGB algorithm also shows a good fit on the calibration decision curves for both the internal test set and the external validation set. Finally, we constructed an online web calculator using the XGB model to help generalize the model and to assist physicians in their decision-making better.

**Conclusion:**

We successfully developed an XGB-based machine learning model to predict liver metastasis from rectal cancer, which was also validated with a real-world dataset. Finally, we developed a web-based predictor to guide clinical diagnosis and treatment strategies better.

## Introduction

Rectal cancer is the eighth most common malignant tumor in the world, with a high mortality rate of about 340,000 lives yearly (1, 2). It has become one of the significant public health problems threatening human health. Patients with rectal cancer often have a poor prognosis due to liver metastasis and whose 5-year survival rate is less than 50% (3, 4). Previous studies have shown that 20-25% of patients with the initial diagnosis of rectal cancer develop liver metastasis. Even after radical resection, rectal cancers still have a 30% probability of liver metastasis (5, 6). Due to the poor prognosis and high prevalence of liver metastasis from rectal cancer, there is still more clinical concern about liver metastasis from rectal cancer (7, 8). Magnetic resonance imaging (MRI) and positron emission tomography/computed tomography (PET/CT) are standard techniques for screening patients with rectal cancer for distant metastasis. However, given the high cost of MRI and the disadvantages of PET-CT radiation damage, it is not recommended for all patients with rectal cancer to be screened for distant metastasis (9, 10). This imposes a high demand for the identification and rationalization of screening people at high risk of liver metastasis from rectal cancer. To address these issues, we used advanced machine learning algorithms to build a predictive model to predict liver metastasis in patients with rectal cancer.

Machine-Learning has become a new type of artificial intelligence that is beginning to be widely used in healthcare data analysis and is a powerful tool for improving clinical strategies (11–15). Machine-learning algorithms can automatically learn from input data to predict outcome values acceptable and identify patterns and trends in the data. A statistical and comprehensive review of machine learning in medical diagnosis by Bhavsar et al. shows that machine learning techniques help medical professionals reduce diagnostic errors, improve healthcare delivery and reduce treatment costs (16). Many scholars have achieved significant breakthroughs by using machine-learning algorithms in colorectal cancer, but machine-learning prediction models for liver metastasis in rectal cancer are not yet available (17, 18). Also, previous studies have limited constructing models only by using public databases, thus limiting the extrapolation of models. Therefore, involving real-world clinical datasets is essential for creating superior predictive models.

This study aims to develop machine-learning models that use clinicopathology features to predict the risk of liver metastasis from rectal cancer and suggest individual prevention strategies to help clinicians make treatment decisions.

## Materials and methods

### Study population

A retrospective analysis of the SEER (Surveillance, Epidemiology, and End Results) database and data from patients admitted to the First Affiliated Hospital of Jilin University and Shanxi Bethune Hospital was conducted. SEER is an authoritative source for cancer statistics in the United States. The Surveillance provides information on cancer statistics to reduce the cancer burden among the U.S. population. The inclusion criteria for the rectal cancer cohort from SEER were demonstrated as follows:(1) the primary pathological diagnosis was rectal cancer, (2) patients without concurrent malignancies, and (3) patients with complete clinical information, including age, gender, race, marital status, histological grade, tumor size, T-stage, N-stage, carcinoembryonic antigen (CEA), diagnostic information, and first site. In addition, the exclusivity criteria were shown as follows: (1) no complete clinical information, (2) another primary neoplastic disease, and (3) unknown liver metastatic status. The inclusion criteria for the external validation set were (1) metachronous liver metastasis (after diagnosis) and (2) patients not undergoing preoperative neoadjuvant therapy. All aspects of the clinical cohort study were approved for inclusion by the Institutional Ethics Committee of the First Hospital of Jilin University and Shanxi Bethune Hospital and were performed in adherence to the Declaration of Helsinki.

### Data collection and data processing

**Figure 1:**
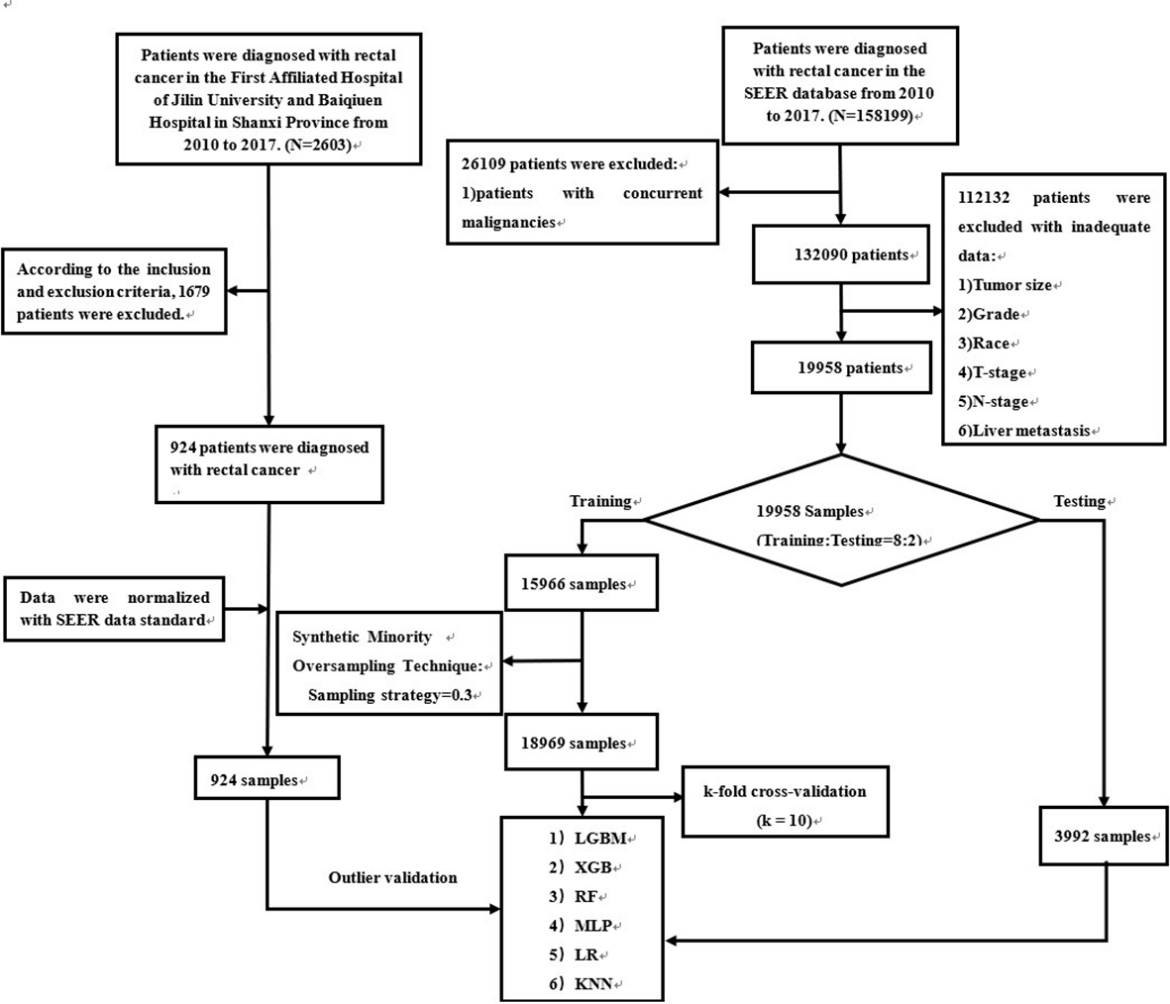
The workflow of the selection procedure for rectal cancer patients. Abbreviations: LGBM, light gradient boosting; XGB, extreme gradient boosting; RF, random forest; LR, logistic regression; KNN, K-nearest neighbor; MLP, multilayer perceptron.

SEER patient data were obtained from “SEER Research Plus Data, 18 Registries, Nov 2020 Sub (2000-2018)” and extracted using SEER * STAT (8.4.0) software. Patients diagnosed with rectal cancer from 2010-2017 were included in this study. Patients with rectal cancer from multiple centers in China were included in the external validation. The entire workflow is demonstrated in detail in **Figure 1**. In addition, patient data from multicenter hospitals were processed according to SEER database standards (**Supplement Table 1**). We transformed the clinical information into numbers to make it easy to compute in the model (**Supplement Table 2**).

### Construction of the predictive model

In this study, we used six machine learning algorithms to predict liver metastasis of rectal cancer, including random forest (RF), Light Gradient Boosting (LGBM), extreme gradient boosting (XGB), multilayer perceptron (MLP), logistic regression (LR) and K-Nearest Neighbor (KNN). RF is a machine learning algorithm that deals with classification and regression problems by building multiple decision tree methods (19). LGBM is a gradient-boosting framework using a tree-based learning algorithm that has been successfully applied to the construction of medical models in recent years (20). XGB is a classical decision tree algorithm applied to classification or regression prediction models (21). MLP is a feedforward neural network model used in different prediction models (22). LR algorithm is a classification algorithm commonly used for dichotomous variables and is widely used in data mining due to its simplicity, parallelizability, and explanatory power (23). KNN algorithm is identified as an essential classification algorithm in the supervised machine-learning domain and is widely used in pattern recognition, data mining, and intrusion detection (24).

Patients from the SEER database were randomly partitioned into a training set and an internal test set using python with a ratio of 8:2. To improve the model’s effectiveness while ensuring the data’s authenticity, we use a synthetic minority oversampling technique (SMOTE) for the SEER database to solve the data imbalance problem (25). The training set was used to build the model, and the internal test set was used for model validation and evaluation. In the training set, k-fold cross-validation (k = 10) is performed, and a grid search is used to find the best combination of parameters. Subsequently, the model performance is initially evaluated in the internal test set. Finally, a rectal cancer cohort from two hospitals was used to validate the model’s generalization capability and efficiency.

### Model performance and feature importance

Model performance is evaluated by area under the curve (AUC), predictive accuracy, sensitivity, specificity, and F1-score. Our primary evaluation metric for machine-learning models is AUC, calculated from ROC curves, which are graphical plots showing the diagnostic power of binary classifiers as their discriminative thresholds change (26). It is also combined with other metrics for a comprehensive evaluation to determine the best model. To further explore the degree of importance of incorporated features in all algorithms, we used the Permutation Importance principle for feature importance analysis of variables. The Permutation Importance approach assesses the effect of model construction on the remaining features by treating an incorporated feature as a random number. If the model performance decreases significantly, the feature is shown to have a more critical role (27). Finally, model performance was then further assessed by calibration curves.

### Interpretability of the model and construction of the web calculator

The Shapley Additive explanation (SHAP) method is used in the article to interpret the constructed model. It allows for the calculation of precisely the contribution of each variable to the final prediction. In addition, each observation in the dataset can be interpreted by a specific set of SHAP values (28). We created a web calculator to make the model clinically easy to use and generalize.

### Statistical analysis

All statistical analyses were performed in Python (version 3.8, Python Software Foundation) and R software (version 4.1.0). All machine learning algorithms were built based on scikit-learn (version 0.24.1). Categorical variables were expressed as totals and percentages, and differences between groups were compared using the χ2 test or Fisher’s exact test. Continuous variables were expressed as median and Standard Deviation (SD), and the Wilcoxon rank sum test was used to compare groups. The results were considered statistically significant when the two-sided P<0.05.

## Result

### Patient components and clinical baseline information

Our study included rectal cancer data from the SEER database, ranging from 2010 to 2017. A total of 152,199 patients with rectal cancer were initially included. Based on established inclusion and exclusion criteria, the final number of patients included in the SEER database was 19,957, 1,712 patients (8.58%) had liver metastasis, and 18,246 patients (91.42%) had no liver metastasis. Outer validation was performed using a total of 924 patients from two centers in China. Patient data from SEER and the two centers in China are presented in **Table 1**.

**Table 1:** Clinical baseline features of SEER and multiple centers hospital database.

Variables	SEER database		Multiple centers
	Training set (N=15966)	Testing set (N=3992)	Outer validation set (N=924)
**Age [mean (SD)]**	61.74 (13.18)	61.72 (13.39)	61.72 (13.31)
Gender, n (%)			
Male	9471 (59.3)	2410 (60.4)	556 (60.2)
Female	6495 (40.7)	1582 (39.6)	368 (39.8)
**Race, n (%)**			
White	12969 (81.2)	3241 (81.2)	0
Black	1324 (8.3)	310 (7.8)	0
Asian or Pacific Islander	1532 (9.6)	402 (10.1)	924
American Indian/Alaska Native	141 (0.9)	39 (1.0)	0
T_stage, n (%)			
T1	2909 (18.2)	695 (17.4)	151 (16.3)
T2	2661 (16.7)	669 (16.8)	140 (15.2)
T3	8870 (55.6)	2236 (56.0)	509 (55.1)
T4	1526 (9.6)	392 (9.8)	124 (13.4)
N_ stage, n (%)			
N0	8604 (53.9)	2159 (54.1)	430 (46.5)
N1	5433 (34.0)	1372 (34.4)	376 (40.7)
N2	1929 (12.1)	461 (11.5)	118 (12.8)
Grade, n (%)			
Grade I	1380 (8.6)	344 (8.6)	53 (5.7)
Grade II	12393 (77.6)	3114 (78.0)	726 (78.6)
GradeI III	1926 (12.1)	471 (11.8)	109 (11.8)
GradeI IV	267 (1.7)	63 (1.6)	36 (3.9)
CEA, n (%)			
Negative	5763 (36.1)	1412 (35.4)	412 (44.6)
Borderline	61 (0.4)	15 (0.4)	75 (8.1)
Positive	4534 (28.4)	1163 (29.1)	242 (26.2)
Unknown	5608 (35.1)	1402 (35.1)	195 (21.1)
Marital, n (%)			
Married	9371 (58.7)	2381 (59.6)	622 (67.3)
Unmarried	4615 (28.9)	1151 (28.8)	231 (25.0)
Other	1980 (12.4)	460 (11.5)	71 (7.7)
**Tumor. size (mean (SD))**	4.37 (3.55)	4.38 (3.86)	4.34 (4.00)
Liver.Met, n (%)			
No	14592 (91.4)	3654 (91.5)	770 (83.3)
Yes	1374 (8.6)	338 (8.5)	154 (16.7)

CEA, carcinoembryonic antigen; Liver_Met, liver metastasis.

Seven clinicopathological factors were included in our established model: age at diagnosis, gender, T-stage, N-stage, CEA, grade of differentiation, and tumor size (**Table 2**). Patients in the SEER database were divided into liver metastasis (LM) and non-liver metastasis groups (NLM). For age at diagnosis, we found that the mean age at diagnosis was significantly more extensive in the NLM group (58.89) than in the LM group (62.01; P<0.001). Notably, the proportion of male patients in the LM group (1119/1712;65.4%) was significantly higher than that in the NLM group (10762/18246; 59%; P<0.001). Unexpectedly, we found no difference between the two subgroups regarding race and married status. According to our assumptions, the LM group showed higher T-stage and N-stage patients (P<0.001). In terms of tumor progression, we found that patients in the LM group (5.70cm) had significantly larger tumor sizes than patients in the NLM group (4.25cm; P<0.001). In addition, we found that patients in the LM group (1070/1712;62.50%) had a higher percentage of positive CEA expression than the NLM group (4627/18246;25.40%;P<0.001).

**Table 2:** Distributions of clinicopathological characteristics in two groups.

Variables	LM	NLM	P value
	N=1712	N=18246	
**Age [mean (SD)]**	58.89 (12.99)	62.01 (13.21)	<0.001
Gender, n (%)			
Male	1119 (65.4)	10762 (59)	<0.001
Female	593 (34.6)	7484 (41.0)	
Race, n (%)			
White	14840 (81.3)	1370 (80.0)	0.080
Black	1466 (8.0)	168 (9.8)	
Asian or Pacific Islander	1774 (9.7)	160 (9.3)	
American Indian/Alaska Native	166 (0.9)	14 (0.8)	
T-stage (%)			
T1	246 (14.4)	3358 (18.4)	<0.001
T2	84 (4.9)	3246 (17.8)	
T3	1067 (62.3)	10039 (55.0)	
T4	315 (18.4)	1603 (8.8)	
N_ stage (%)			
N0	486 (28.4)	10277 (56.3)	<0.001
N1	811 (47.4)	5994 (32.9)	
N2	415 (24.2)	1975 (10.8)	
Grade			
Grade I	89 (5.2)	1635 (9.0)	<0.001
Grade II	1298 (75.8)	14209 (77.9)	
Grade III	280 (16.4)	2117 (11.6)	
Grade IV	45 (2.6)	285 (1.6)	
CEA			
Negative	232 (13.6)	6943 (38.1)	<0.001
Borderline	3 (0.2)	73 (0.4)	
Positive	1070 (62.5)	4627 (25.4)	
Unknown	407 (23.8)	6603 (36.2)	
Marital			
Married	965 (56.4)	10787 (59.1)	0.063
Unmarried	516 (30.1)	5250 (28.8)	
Other	231 (13.5)	2209 (12.1)	
**Tumor size (mean (SD))**	4.25 (3.46)	5.70 (4.84)	<0.001

CEA, carcinoembryonic antigen; LM, liver metastasis; NLM, non-liver metastasis.

### Correlation of variables and feature importance of prediction

**Figure 2:**
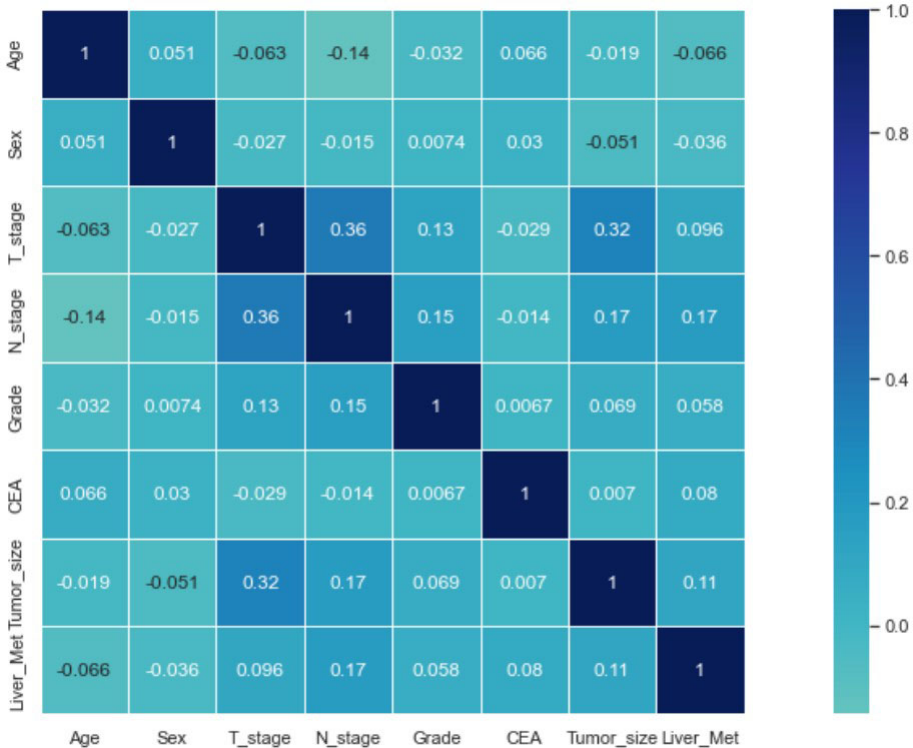
Results of the correlation heatmap between all variables. The graph shows the correlations between all variables.

**Figure 3:**
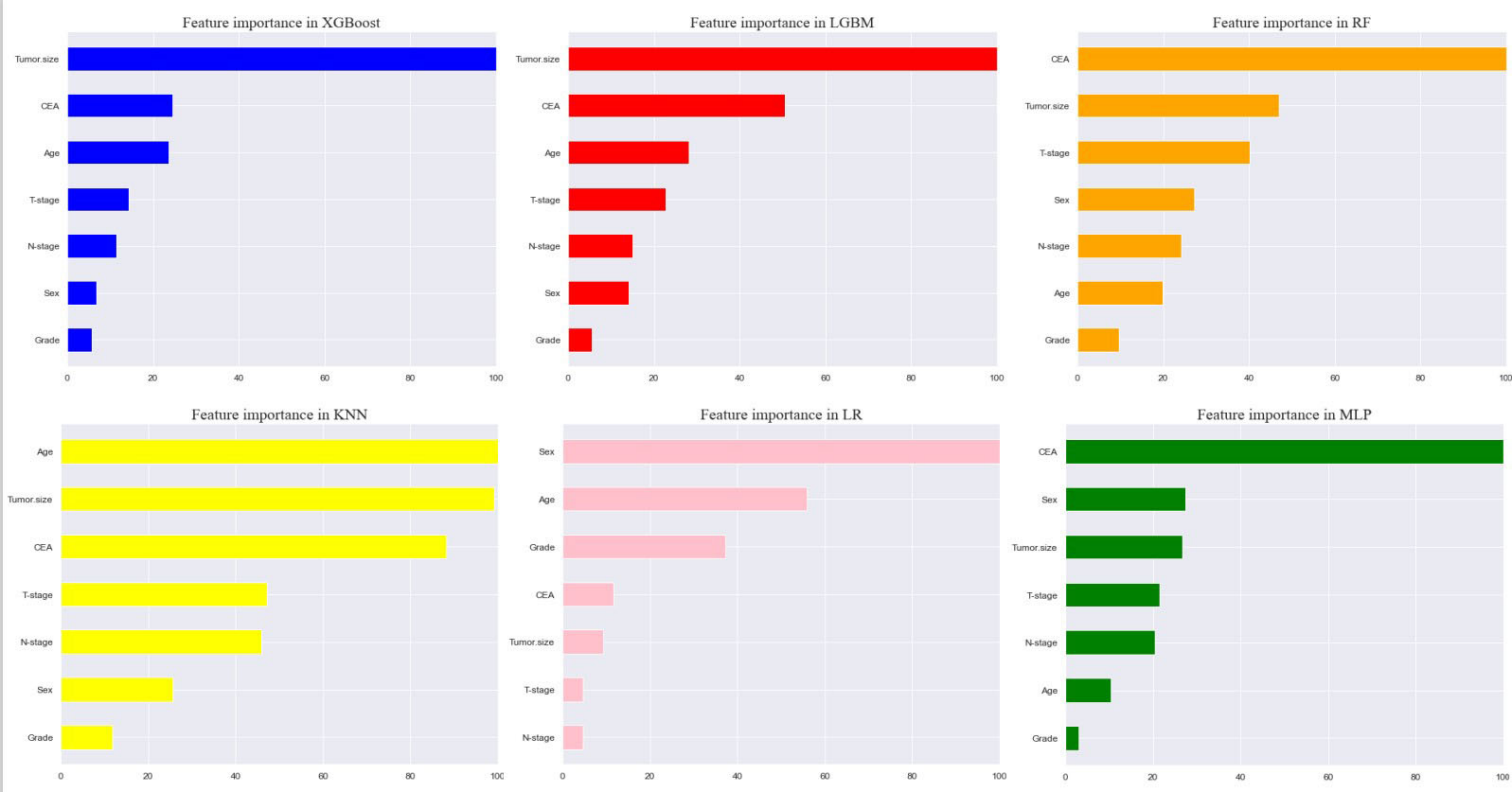
Feature importance of six models. The plot shows the ranking of six models’ relevant volume of features. Abbreviations: LGBM, light gradient boosting; XGB, extreme gradient boosting; RF, random forest; LR, logistic regression; KNN, K-nearest neighbor; MLP, multilayer perceptron.

Seven selected variables were analyzed according to the spearman correlation test, and the heatmap results showed no significant correlation between the variables, and multicollinearity is unlikely (**Figure 2**). The importance ranking of features in the six machine-learning models is shown in **Figure 3**. The relative importance of the variables in the six machine learning models was analyzed using the permutation importance principle. Although the six machine learning algorithms ranked differently regarding the importance, CEA and tumor size ranked in the top two of most models. They may have a more critical predictive role for rectal cancer liver metastasis. In contrast, sex and grade ranked lower but contributed to rectal cancer liver metastasis. In the XGB model, the importance of features were ranked in descending order by tumor size, CEA, age, T-stage, N-stage, sex, and grade.

### Model performance and model explainability

**Figure 4:**
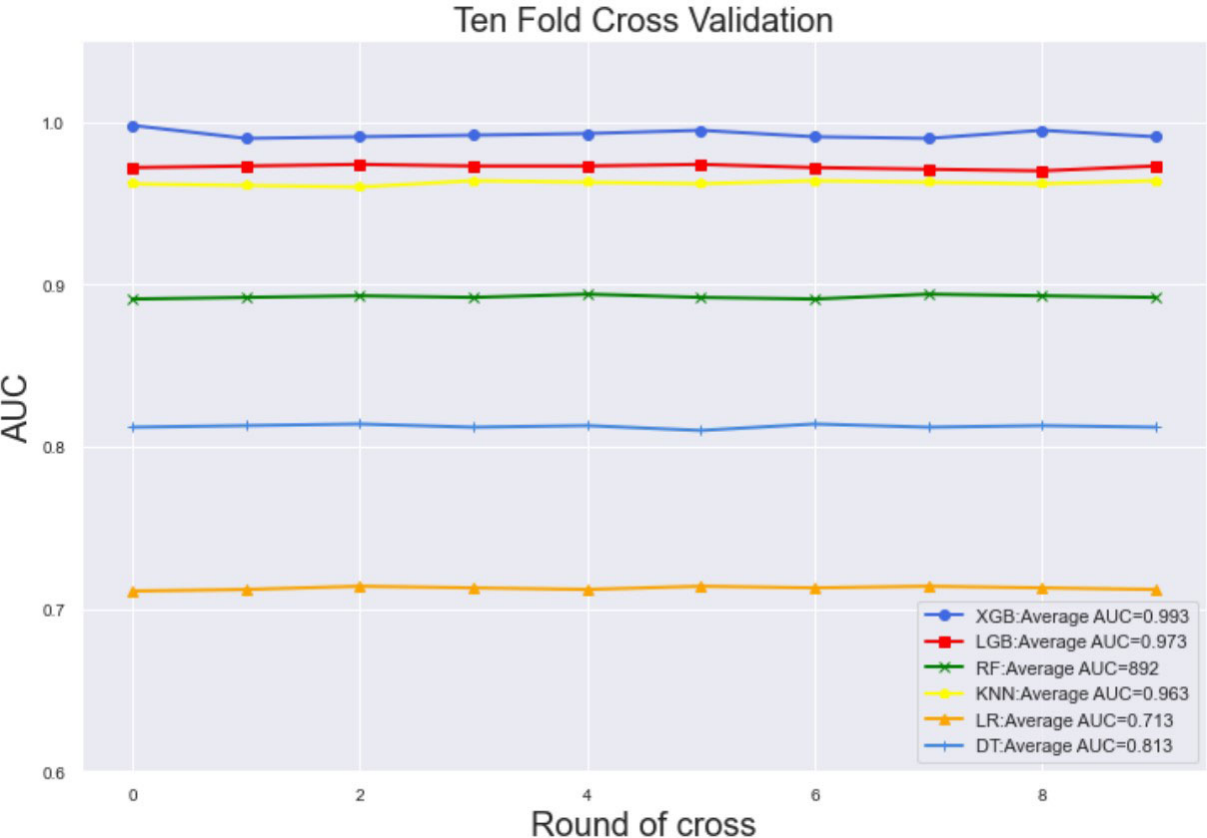
The training set has ten-fold cross-validation results of different machine learning models. Abbreviations: LGBM, light gradient boosting; XGB, extreme gradient boosting; RF, random forest; LR, logistic regression; KNN, K-nearest neighbor; MLP, multilayer perceptron.

**Figure 5:**
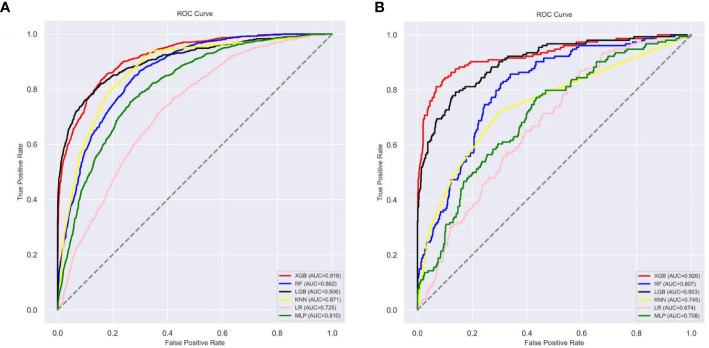
The roc curves of different machine learning models: **(A)** The roc curves of other machine learning models in the internal test set; **(B)** The roc curves of other machine learning models external test set. Abbreviations: LGBM, light gradient boosting; XGB, extreme gradient boosting; RF, random forest; LR, logistic regression; KNN, K-nearest neighbor; MLP multilayer perceptron; roc, receiver operating characteristic.

**Figure 6:**
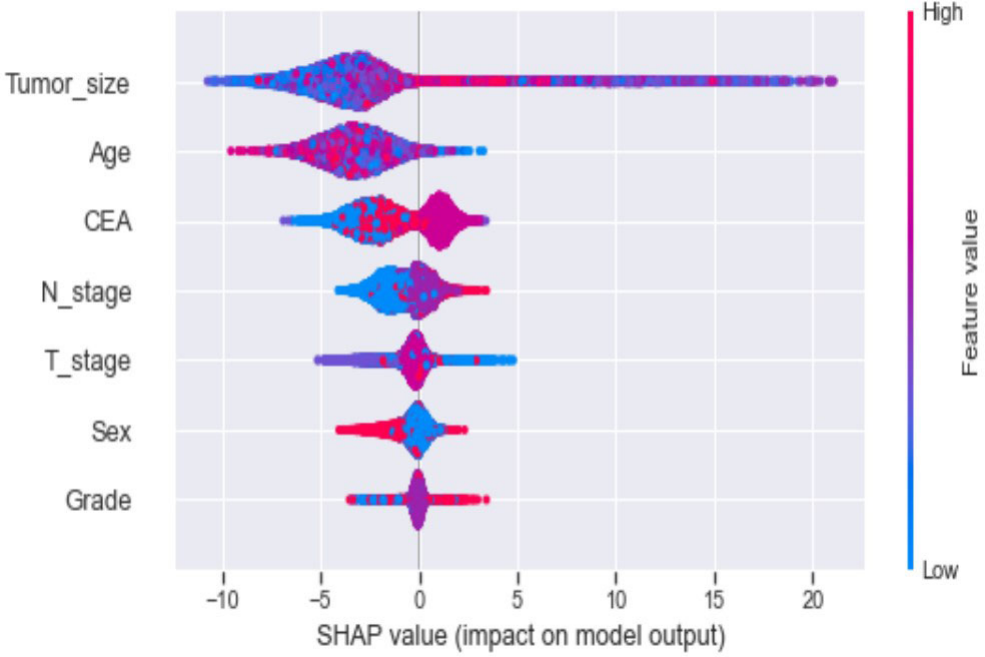
The Shapley Additive explanation (SHAP) values.

Ten-fold cross-validation of the training set data using six machine learning algorithms shows that the XGB model has an average AUC value of 0.993, indicating the best predictive power among all models (**Figure 4**). In the internal test set, XGB obtained an AUC of 0.918, an accuracy of 0.884, a sensitivity of 0.721, and a specificity of 0.787(**Figure 5**; **Table 3**). In the outer test set, the XGB model also showed excellent performance with an AUC of 0.926, an accuracy of 0.919, a sensitivity of 0.740, and a specificity of 0.765 (**Figure 5**; **Table 3**). The calibration curves for the internal and outer validation sets also demonstrate that the model has a reasonable degree of fit (**Supplementary Table 3**). Finally, to detect positive and negative correlations between characteristics and liver metastasis of rectal cancer, we used SHAP to reveal risk factors for liver metastasis of rectal cancer. The related results are presented in **Figure 6**.

**Table 3:** Comparison prediction performances of different models for liver metastasis.

	model	AUC	Accuracy	Sensitivity	Speciality	F1-score
Internal test set						
	XGB	0.918	0.884	0.721	0.787	0.733
	LGBM	0.906	0.879	0.622	0.746	0.695
	RF	0.862	0.824	0.460	0.644	0.537
	KNN	0.871	0.840	0.561	0.665	0.608
	LR	0.725	0.774	0.081	0.440	0.137
	MLP	0.810	0.806	0.390	0.591	0.470
Outer validation set						
	XGB	0.926	0.919	0.740	0.765	0.752
	LGBM	0.903	0.903	0.610	0.758	0.676
	RF	0.807	0.825	0.318	0.462	0.377
	KNN	0.745	0.845	0.300	0.568	0.391
	LR	0.674	0.824	0.032	0.263	0.058
	MLP	0.708	0.803	0.156	0.316	0.209

LGBM, light gradient boosting; XGB, extreme gradient boosting; RF, random forest; LR, logistic regression; KNN, K-nearest neighbor; MLP, multilayer perceptron; AUC, area under the curve.

### Web predictor

**Figure 7:**
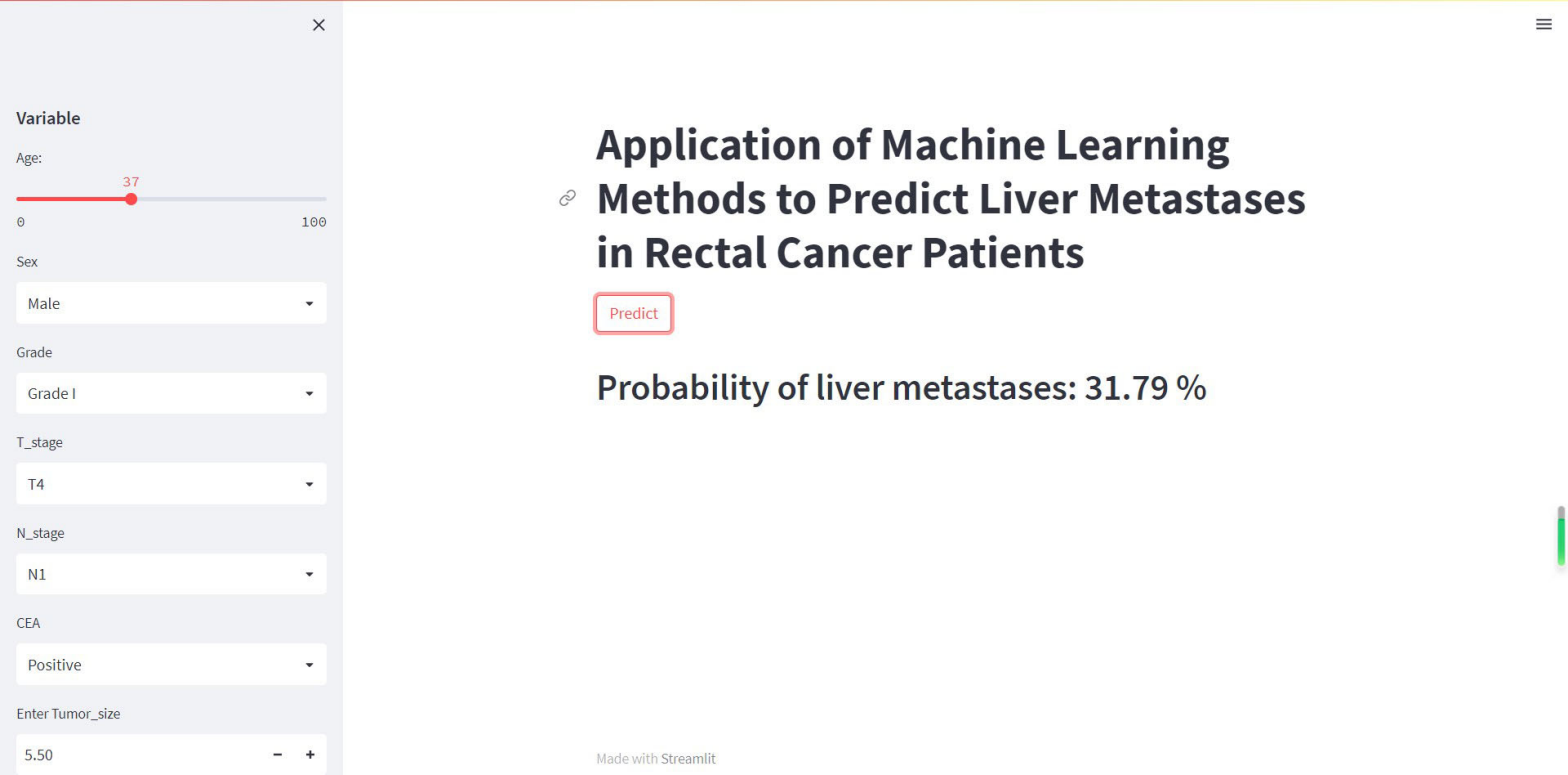
The machine learning model-based web predictor for predicting liver metastasis in rectal cancer patients.

Although XGB has shown excellent predictive ability in liver metastasis from rectal cancer, it is intricate and complex, which is not conducive to clinical dissemination. Therefore, this study developed an online web calculator for predicting liver metastasis risk in patients with rectal cancer. The calculator can be easily extended clinically and only requires inputting patient clinicopathological information to derive the probability of obtaining liver metastasis in patients with rectal cancer while stratifying patients into high and low risk. (https://share.streamlit.io/woshiwz/rectal_cancer/main/rectal.py) (**Figure 7**).

## Discussion

In this study, we constructed a model for predicting liver metastasis from rectal cancer using popular machine-learning algorithms combining seven clinical and pathological features. To the best of our knowledge, this study is the first article on the prediction of liver metastasis in rectal cancer combined with machine learning algorithms while using real-world data for outer validation. The AUC performance of these models is mostly more extensive than 0.85 (**Figure 5**; **Table 3**), while the accuracy is mostly above 0.80 (**Figure 5**; **Table 3**); therefore, we believe that the developed models are robust and reliable while allowing for more significant clinical benefits. Comparing the prediction performance of six machine-learning algorithms, we found that the model based on the XGB algorithm performed the best. Finally, to make the model clinically applicable and generalizable, we developed an online web calculator based on the XGB model to calculate the probability of liver metastasis from rectal cancer and thus screen patients at high risk for liver metastasis.

In addition, machine learning’s clinical importance is reflected in identifying important risk factors associated with liver metastasis from rectal cancer (29). In this study, tumor size, CEA, age, T-stage and N-stage were critical for liver metastasis from rectal cancer based on XGB algorithms’ ranking of feature importance. In the present study, tumor size showed the best predictor in numerous models. Large tumor size indicates a long growth cycle, leading to a more proliferative and aggressive state of tumor cells, which increases liver metastasis (29, 30). CEA is the standard tumor marker on colorectal cancer cell membranes and embryonic mucosal cells. An increasing number of studies have shown that pretreatment CEA levels are considered to be associated with tumor stage and metastasis, with studies showing that CEA is elevated in 45% of patients with stage B and 76% of patients with distal metastasis (31–34). Therefore, it is unsurprising that the preoperative serum CEA levels of patients with rectal cancer liver metastasis in this study were higher than those of non-liver metastasis. Although the proportion of young rectal cancer patients is increasing yearly, there is convincing evidence that younger people can achieve better outcomes than older patients (35).Interestingly, this study showed that the younger the patient, the higher the risk of liver metastasis from rectal cancer. The reason may be that younger patients’ tumor cells often undergo mismatch repair and thus upregulate the tumor’s aggressiveness (36–38). T-stage is an essential indicator of tumor progression and positively correlates with tumor metastasis in most tumors. Our results demonstrate that patients with rectal liver metastasis have a more advanced T-stage than those with non-rectal liver metastasis. Considerable research suggests that the reason may be due to the progressive increase in lymphatic vessels from the mucosal to the plasma layer of the rectal wall structure and the abundance of lymphatic reflux, which in turn increases the risk of liver metastasis from rectal cancer (39, 40). Several studies have shown that patients with regional lymph node metastasis are likelier to develop liver metastasis from rectal cancer (41–43). The liver is one of the organs with the most abundant lymphatic tissue in the body. Therefore, tumors are more likely to metastasize when local lymph nodes are metastatic. Although gender and differentiation grades did not perform well in the XGB model in this study, they still played a role in model construction. Tang et al. found that men and poorly differentiated patients were more likely to develop liver metastasis from rectal cancer, in concurrence with the results of this study (44, 45). Our model sufficiently integrates various risk factors that could affect liver metastasis from rectal cancer and achieves outstanding predictive performance.

Most traditional statistical methods are based on parametric regression models that assume a linear relationship between variables and outcomes (29, 46). However, we should know that most variables and results are more than just linearly related. With the rapid development of artificial intelligence, machine-learning algorithms play an increasing role in tumor diagnosis and prognosis assessment. Machine-learning algorithms have many advantages, including preventing overfitting and handling unbalanced data (47). In this study, XGB performed better than other algorithms because it added a regular term to the objective function to control the complexity of the model and avoid overfitting while supporting column sampling to enhance the stability of the model (21, 48).

Until now, only surgical resection has proven to be a curative treatment for patients with early resectable rectal cancer liver metastasis (49). Early systemic chemotherapy may improve prognosis and increase median survival in patients with undetectable rectal cancer liver metastasis (50). Combining all these results, we believe that further use of the rectal cancer liver metastasis model will aid clinical decision-making and improve the current treatment status.

The strengths of this study are the use of large-scale data from the SEER database for modeling and the use of real-world clinical data for outer validation, which prevents the overfitting of the data and ensures extrapolation of the model. However, this study also has some limitations. Firstly, the study is a retrospective study, and there may be selection bias in the sample selection. Secondly, there are limitations in the data available in the SEER database, such as the unavailability of crucial information on chemotherapy regimens, radiotherapy doses, and vascular infiltration, which limits the predictive value of our model. Finally, only two outer validation sets were used to validate the model, and further efforts are needed to validate the model’s performance on a more diverse population. These reasons may lead to a limited verification effect of our model.

For future work, we will focus on prospective and diverse population validation of the models to verify their performance and stability. These models are then expected to be integrated into applications that assist clinicians in medical decision-making. This can be a step toward a semi-autonomous diagnostic system that can assist clinicians in making individualized diagnoses of liver metastasis for patients with rectal cancer.

## Conclusion

In conclusion, we developed a prediction model for liver metastasis from rectal cancer, which uses machine-learning algorithms to predict liver metastasis from rectal cancer. A web calculator has also been designed to facilitate the screening of patients at high risk of liver metastasis from rectal cancer by inputting some parameters, which may help physicians to individualize the treatment of rectal cancer patients.

## Data availability statement

The original contributions presented in the study are included in the article/**Supplementary Material**. Further inquiries can be directed to the corresponding author.

## Ethics statement

Written informed consent was obtained from the individual(s), and minor(s)’ legal guardian/next of kin, for the publication of any potentially identifiable images or data included in this article.

## Author contributions

BQ and QW designed the study. BQ, XQ, and XS conducted data analysis. BQ conceived the project and wrote the manuscript. QW revised and approved the paper. All authors contributed to the article and approved the submitted version.
