# Supplementary material for: Application of machine learning techniques in real-world research to predict the risk of liver metastasis in rectal cancer
Source: Front Oncol. 2022 Dec 20;12:1065468. doi: 10.3389/fonc.2022.1065468 (PMC9807609; doi:10.3389/fonc.2022.1065468)
Supplement: Supplementary file 1 [file Table_1.docx]

**Supplement Table S1. Normalization standards of clinical data in outer validation set.**

| **Clinical data** | **Normalization Standard** |
| --- | --- |
| **Basic demographic data** |  |
| Age | Age at diagnosis. |
| Marital status | Marital status at diagnosis |
| Sex | Sex |
| Race | Race |
| **Tumor information** |  |
| Tumor size | the largest diameter of the primary tumor. |
| TNM stage | According to AJCC 7th edition. |
| Tumor grade | Well differentiated, Moderately differentiated, Poorly differentiated, and Undifferentiated. |
| **Laboratory index** |  |
| CEA | Positive (Nonsmoker: >2.5ng/ml, Smoker: >5ng/ml), |
|  | Negative (Nonsmoker: <2.5ng/ml, Smoker: <5ng/ml). |
|  | Borderline (Nonsmoker: 2.5ng/ml, Smoker: 5ng/ml), |

**Abbreviation:** AJCC, American Joint Committee on Cancer; CEA, carcinoembryonic antigen.
