# Supplementary material for: Application of machine learning techniques in real-world research to predict the risk of liver metastasis in rectal cancer
Source: Front Oncol. 2022 Dec 20;12:1065468. doi: 10.3389/fonc.2022.1065468 (PMC9807609; doi:10.3389/fonc.2022.1065468)
Supplement: Supplementary file 2 [file Table_2.docx]

Table S2 References for property values of clinical features in models.

| **Variables** | **Property Values** |
| --- | --- |
| **Sex:** |  |
| Male | **1** |
| Female | **2** |
| **Race:** |  |
| White | **1** |
| Black | **2** |
| Asian or Pacific Islander | **3** |
| American Indian/Alaska Native | **4** |
| **T_stage:** |  |
| T1 | **1** |
| T2 | **2** |
| T3 | **3** |
| T4 | **4** |
| **N_stage:** |  |
| N0 | **1** |
| N1 | **2** |
| N2 | **3** |
| **Grade:** |  |
| Well differentiated; Grade I | **1** |
| Moderately differentiated; Grade II | **2** |
| Poorly differentiated; Grade III | **3** |
| Undifferentiated; anaplastic; Grade IV | **4** |
| **Marital_status:** |  |
| Married | **1** |
| Unmarried | **2** |
| **CEA:** |  |
| Negative | **1** |
| Borderline | **2** |
| Positive | **3** |
| Unknown | **4** |
| **Liver_Met:** |  |
| No | **0** |
| Yes | **1** |
