# Supplementary figures and images for: Application of machine learning techniques in real-world research to predict the risk of liver metastasis in rectal cancer
Source: Front Oncol. 2022 Dec 20;12:1065468. doi: 10.3389/fonc.2022.1065468 (PMC9807609; doi:10.3389/fonc.2022.1065468)

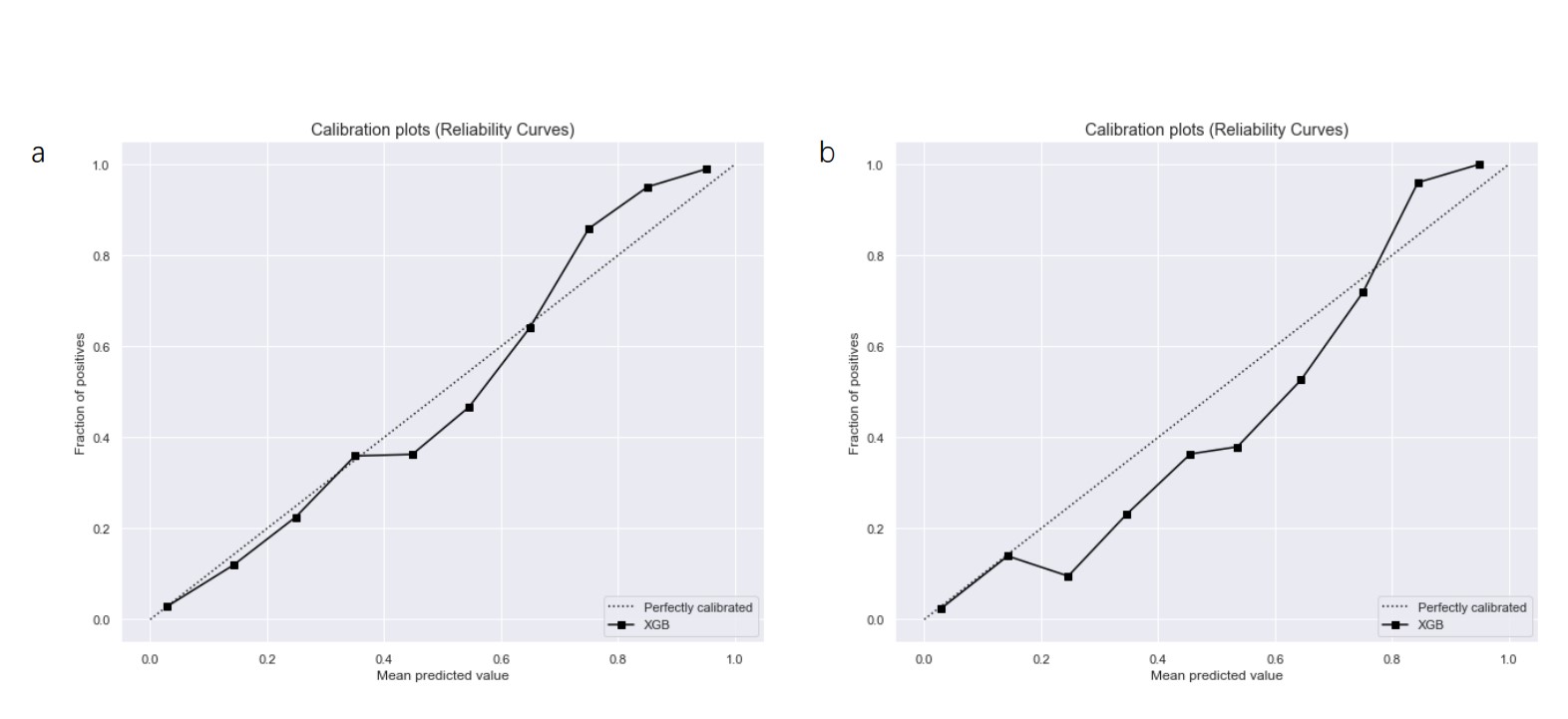
Supplement table 3:The calibration curves of the internal test set(a) and outer validation set(b)

Supplement: Supplementary file 3 [file Table_3.docx]
